# Draft Genome Sequence of an *Escherichia coli* O8:H19 Sequence Type 708 Strain Isolated from a Holstein Dairy Cow with Metritis

**DOI:** 10.1128/genomeA.00261-16

**Published:** 2016-04-07

**Authors:** Amber Ginn, Zhengxin Ma, Klibs N. Galvao, KwangCheol Casey Jeong

**Affiliations:** a Emerging Pathogens Institute, University of Florida, Gainesville, Florida, USA; b Department of Animal Sciences, Institute of Food and Agricultural Sciences, University of Florida, Gainesville, Florida, USA; c Department of Large Animal Clinical Sciences, College of Veterinary Medicine, University of Florida, Gainesville, Florida, USA; d D. H. Barron Reproductive and Perinatal Biology Research Program, University of Florida, Gainesville, Florida, USA

## Abstract

We present here the genome sequence of *Escherichia coli* O8:H19 strain KCJ852, belonging to multilocus sequence type (MLST) 708, isolated from the uterus of a cow with a bovine postpartum uterine infection known as metritis. Genomic investigation of KCJ852 will help us understand its virulence potential.

## GENOME ANNOUNCEMENT

Pathogenic *Escherichia coli*, which causes bovine postpartum uterine infections, such as metritis (1), is termed intrauterine pathogenic *E. coli* (IUPEC) or endometrial pathogenic *E. coli* (EnPEC) (2). *E. coli* O8:H19 strain KCJ852, from multilocus sequence type 708 (ST708), was isolated from a Holstein dairy cow with metritis and was further classified as an IUPEC strain at the University of Florida Dairy Research Unit, Gainesville, FL. The sequencing of this genome provides further genomic characterization for this pathogen specifically related to colonization in the uterus. KCJ852 encodes characteristic multiple fimbriae, iron acquisition systems, and autotransporter proteins (type 5 secretion system), as well as flagella, similar to another EnPEC isolate, MS499 (3). In addition, through a comparison of specific virulence genes found in both KCJ852 and MS499, we identified two unique genes in KCJ852 known as *orf*68 and *gspF*, which are associated with adherence and the type II secretion system, respectively. Comparative genomics of similar IUPEC or EnPEC strains belonging to ST-708, as well as other *E. coli* isolates not classified as IUPEC strains but having close phylogenetic distance to the ST-708 clade, will provide insight into the evolution of this pathogen.

KCJ852 was cultured overnight at 37°C in Luria broth, and pure genomic DNA was extracted using the DNeasy blood and tissue kit (Qiagen), according to the protocol for Gram-negative bacteria, providing high-quality DNA. DNA quantification was performed using the Qubit fluorometer high-sensitivity double-stranded DNA assay, with a dilution of 0.2 ng per µl used as input DNA for tagmentation with Illumina’s Nextera XT DNA library sample preparation kit. Libraries were sequenced using the benchtop Illumina MiSeq, with a 2 × 250-bp, 500-cycle cartridge. The average read quality per base was 37, with genome coverage of 21×. FastQ files were checked for quality control using FASTQC (http://www.bioinformatics.babraham.ac.uk/projects/fastqc/), and reads were trimmed for quality and length using sickle (4), with the length parameter set to 50 and quality set to 30. *De novo* assemblies were performed with SPAdes (5) using a k-mer list of 21, 33, 55, 77, 99, and 127. Three hundred three contigs were created from 301 scaffold sequences, with all contigs <200 bp removed. The resulting assembled genome produced 4,725,662 bp, with 53% mean G+C content per sequence. PATRIC annotation (6) provided 24 invasion genes and numerous other virulence genes that facilitated us with an in-depth analyses of genes present in both other IUPEC and non-IUPEC strains of close genetic distance, which will reveal virulence characteristics specific to its lineage.

### Nucleotide sequence accession numbers.

This whole-genome shotgun project has been deposited at DDBJ/ENA/GenBank under the accession no. LSFT00000000. The version described in this paper is version LSFT01000000.
